# Understanding the Joint Impacts of Cognitive, Social, and Geographic Proximities on the Performance of Innovation Collaboration Between Knowledge-Intensive Business Services and the Manufacturing Industry: Empirical Evidence From China

**DOI:** 10.3389/fpsyg.2022.862939

**Published:** 2022-04-08

**Authors:** Ting Zhao, Meng Yang, Zhijuan Cao, Xiang Wang

**Affiliations:** ^1^School of Economics, Zhejiang Gongshang University, Hangzhou, China; ^2^Business School, Yunnan University of Finance and Economics, Kunming, China; ^3^School of Statistics and Mathematics, Yunnan University of Finance & Economics, Kunming, China; ^4^Yunnan College of Tourism Vocation, Kunming, China

**Keywords:** KIBS-manufacturing collaboration, geographic proximity, cognitive proximity, social proximity, innovation performance

## Abstract

Most previous empirical studies just addressed the influence of geographical proximity on interactive learning regarding the collaboration between knowledge-intensive business service (KIBS) and manufacturing industries. Drawing upon the social cognitive and knowledge-based perspective, this study bridged the research gaps by investigating the joint effects of geographical proximity and two representative non-geographic-proximities (i.e., cognitive proximity and social proximity) in fostering manufacturing firms’ innovation performance. In terms of the empirical analysis, we applied a research sample that involves the data of various manufacturing industries in 260 cities of China from 2003 to 2014 to test the corresponding hypotheses. Additionally, the Spatial Durbin Model (SDM) was adopted and the research findings showed that: (i) the geographic and social proximity significantly promote the knowledge transfer from KIBS to manufacturing firms, which further improves the innovation performance of the latter. However, the effect of cognitive proximity presents insignificant; (ii) the interactive effect of geographic and social proximity was positively associated with the innovation collaboration between KIBS and manufacturing firms; (iii) although the individual effect of cognitive proximity was insignificant, when it interacted with geographic or social proximity, the joint effects were proved to promote the innovation performance of manufacturing firms. This study extends our understanding pertaining to the influencing mechanism of proximity for KIBS and the innovation process. The findings proved that geographic and social proximity are two imperative facilitators of knowledge-creating collaboration, highlighting their indispensable role in moderating and mediating the knowledge transfer of KIBS as well as the innovation performance of manufacturing firms. Notably, cognitive proximity is contingent upon geographic and social proximity on its positive effects on the innovation performance for KIBS and their clients’ collaboration.

## Introduction

In today’s higher-order service-based economy era, knowledge-intensive business service (KIBS) has been well documented in its vital role in innovation systems. KIBS detects practical needs, defines product specifications, and provides themselves a place to exchange and communicate high-level and customized knowledge with their clients (Gallouj and Windrum, 2009; Landry et al., 2012; Pace and Miles, 2019). Most literature has proved KIBS as a critical external knowledge base, knowledge brokerage, as well as important and successful contributor to the generation and diffusion of innovation (Mas-Verdú et al., 2011; Santos-Vijande et al., 2013). It is especially highlighted that the innovation development of the manufacturing industry is closely related to KIBS given that they are primary service clients of KIBS (Ciriaci and Palma, 2016; Seclen and Barrutia, 2018; Zhao et al., 2021).

Nevertheless, despite the advantages of KIBS for promoting innovation performance, it is still a challenging and uncertain task for most organizations to acquire innovative knowledge through effective communication channels, which make full use of the potential benefits derived from the desired service and even foster an efficient collaboration with KIBS, because of the complexity and uncertainty to manage service innovation while dealing with various problems, which addresses multi-dimensional and organization-wide challenges for managers who are responsible for overall deployment and implementation (Kindstrom et al., 2013). Therefore, scholars are encouraged to conduct further investigation on the various factors affecting the interactive learning between KIBS and their clients.

Among the factors affecting the innovation performance that are built upon a service supply-demand relationship, geographical proximity is highlighted in the research regarding service innovation and inter-organizational collaborations. In this vein, fruitful research has focused on the importance of geographical proximity on KIBS and their innovation activities (Nachum and Keeble, 2002; Wood, 2002; Serrano, 2019), while these studies have reached a consensus on the proximity between the actors within an open innovation economy, namely, “right over there,” matters a lot (Gertler, 2003; Storper and Venables, 2003). However, extant scholars have recently questioned the relationship between geographical proximity and innovation, arguing that a firm’s knowledge base, even in the most advanced industrial clusters, is not exclusively associated with its local networks. Hence, it calls for future studies regarding the implications of proximity on innovation from a novel perspective. First, given that proximity is not a simply spatial phenomenon, the concept of proximity is not limited to geographical dimension. Scholars clarified that there are different kinds of non-geographic proximities, which are further divided into cognitive, social, institutional proximity (Boschma, 2005; Knoben and Oerlemans, 2006), etc. In particular cases, non-geographic proximities are more impressive than geographical proximity. Second, the efficiency of interactive learning and knowledge spillover between collaboration partners are largely determined by the interactions of different dimensions of proximity (Torre, 2008; Broekel, 2012; Hansen, 2015).

However, there is still insufficient discussion concerning how various dimensions of proximity and their complex interrelationships on KIBS firms’ knowledge-related activities. There remains a critical research issue that deserves further exploration regarding how the intersections of proximities influence the innovation performance of the manufacturing clients of KIBS, which are regarded as their major customers through innovation collaborations. Specifically, we believe that geographical proximity, social proximity (SP), and cognitive proximity are the three most representative dimensions of proximities. In terms of the channels of communication, extant studies mostly focused on geographical proximity, claiming that there will be a certain space limitation regarding knowledge spillovers. Nevertheless, it is noted that knowledge flows based on a spatial network in which different kinds of agents with various knowledge bases and portfolios voluntarily exchange knowledge and resources through official or unofficial interactions. In this way, given that the influencing effects pertaining to social, institution, technology, and organizations exist in the certain space, we reckoned that the above mentioned three dimensions of proximities exert supplementary and reinforcing effects to the service innovation of KIBS, which is significant to incorporate them for further empirical analysis. On the other hand, we have witnessed an increasing tendency that manufacturing firms strategically involve collaborations with KIBS firms in their various kinds of innovation processes, so as to promote their productivity and innovation capability in a knowledge-based economy. However, it is evident that firms are facing complex tasks in an evolutionary economic environment. Considering that specialized and diversified knowledge coexist, an efficient inter-organizational collaboration pertaining to knowledge creation is promoted, which is essential for manufacturing firms to upgrade their existing knowledge and technology to improve their innovation performance for the long run.

This study set out to solve the following three issues: (i) Which dimensions of proximity are critical to knowledge transfer between KIBS and their manufacturing clients? Do the effects of geographic and non-geographic proximities vary in affecting the latter’s innovation performance? (ii) How are geographical proximity and its non-geographic counterparts interrelated and influence the collaborative innovation performance between KIBS and its manufacturing clients? (iii) What is the effective spatial range regarding the individual and interaction effects of different proximity dimensions on the innovation performance of the collaboration between KIBS and manufacturing industries? In this regard, we believe that this study is of great theoretical significance to advance service innovation and inter-organizational collaboration literature by elaborating on how various dimensions of proximity, individually and jointly, influence the knowledge spillovers from KIBS to their manufacturing clients, thereby affecting innovation effects of KIBS-manufacturing collaborations. In terms of the empirical test, our analysis adopted the Knowledge Production Function (KPF) framework. We specified the general form of the econometric model as a Cobb-Douglas function, where the innovation output (INN) is a function of KIBS input which is multiplied by proximity factors from other regions, and R&D expenditures a set of control variables. A big panel data set is utilized referring to more than 300,000 manufacturing firms in China, which involved data of patent applications, and data pertaining to KIBS in 260 cities in China from 2000 to 2014. The spatial econometric techniques allow us to model the role of knowledge spillovers transmitted through the aforementioned different proximity channels.

Subsequent parts of this study are organized as follows. First, we elaborate on a systematic literature review and relevant theoretical background of different dimensions of proximity, especially for how these proximities accelerate the knowledge spillover and innovation performance of KIBS manufacturing clients through collaboration. Second, the effects of the intersections between the three dimensions of proximities on innovation and knowledge spillover are articulated and justified. Hence, we proposed this study’s hypotheses. Following this, we describe the research model, variables, empirical analysis, and then report the empirical results. Finally, we outline the theoretical and practical implications of our proposed new concepts and discuss avenues for future research.

## Theoretical Basis and Research Hypotheses

### Geographical Proximity and Innovation

Geographical proximity is the dimension of proximity that has been long highlighted by the economic geographers, who focus on how geographical proximity improves knowledge exchange and collaboration’s performance of actors. It is especially true that geographical boundary strongly influences the spatial diffusion of tacit knowledge (Cassar and Nicolini, 2008). When there is a high level of geographical proximity, it is much easier for actors to share and acquire new knowledge. The contribution of geographical proximity can be attributed to frequent face-to-face communication between actors, increasing the possibility of desirable matching (Duranton and Puga, 2004), and more conducive to collective learning (Paier and Scherngell, 2011).

The above advantages of geographical proximity are strengthened in specific industrial clusters, cities, and regions where firms, suppliers, service providers, and diverse industries are highly concentrated (Caragliu et al., 2016). Considering the externality and complementarity, actors interacting with each other enables a larger scope of a collaboration system in which various sets of organizations in different industries build up production links of products and services. In this regard, it is conducive for actors that have general localized knowledge to acquire new knowledge in a specific region (Papanastassiou and Pearce, 1994; Zanfei, 2000). Nevertheless, the agglomeration environment promotes the interactive learning abilities of actors, which facilitates the dynamic process of new innovative strategies (Wood, 2002).

### Non-geographical Proximity and Innovation

As discussed above, the critical role of proximity for innovation, the impact of non-geographical proximity on knowledge exchange and inter-organizational cooperation has long been neglected or mixed up with geographical proximity, while in the last two decades, the rise of studies represented by “scholars of proximity dynamics” has distinguished and clarified various dimensions of proximity from the geographic one (e.g., social proximity, cognitive proximity, institutional proximity, and organizational proximity) (Boschma, 2005), and prove that non-geographical proximity is more likely to affect interactive learning, the formation of knowledge cluster (Lazzeretti and Capone, 2016), and innovation collaboration positively (Knoben and Oerlemans, 2006; Capaldo and Petruzzelli, 2014). The recent research breakthroughs have inspired the new research enthusiasm about how non-geographical proximity affects the knowledge diffusion and the innovation process.

### Social Proximity and Innovation

Social proximity usually refers to a friendly atmosphere (Oerlemans and Meeus, 2005), in which actors share interpersonal relationships, social networks, and the same beliefs or follow unified institutions of the community (Torre and Rallet, 2005).

Social proximity will be strengthened through friendship, trust, past experiences of cooperation, repeated communication, reputation feedback (Balland, 2012), as well as interpersonal emotional regulations. In cultures that place a high value on social interconnectedness (Liddell and Williams, 2019). The concept of SP is closely related and highly overlapping with organizational proximity and institutional proximity (Heringa et al., 2014), which indicate a process of individual socialization within a firm, usually involving stable partner relations with SP within a unified institutional framework (Hung et al., 2020). So, in empirical study, it is very difficult to distinguish a very clear scope and measure among these three proximity dimensions. To simplify the analysis, we integrate these two dimensions of proximity into SP.

Fruitful studies have proved that trust-based social relations are conducive to interactive learning of tacit knowledge and innovation performance (Breschi and Lissoni, 2005; Sorenson et al., 2006). It is explained that when there are some specific commons primarily cultural, institutional background (e.g., habits, norms, language, etc.) and previous cooperation experience between actors, there will be a relatively stable basis for trust and confidence building, which is of great importance for decreasing the coordination costs between actors and therefore, tacit knowledge creation and dissemination are supported by the common social and institution environments (Pisoni, 2016).

As SP covers all connections within different actors (Janssen et al., 2020), which constitutes a crucial channel for information exchange on technological developments and emerging market opportunities (Owen-Smith and Powell, 2004), specifically, the source capital brought by social networks which has derived from the social closeness of different actors is a precise key prerequisite for the search for complementary knowledge (Laursen et al., 2012), whereas the embeddedness of a firm in its social network contributes to developing an organization’s learning capacity, resource sharing, cooperation, and adaptation, thereby improving its innovative and economic results (Uzzi, 1996). It has been proved by extant empirical studies that a firm’s social network significantly impacts its performance (Ahuja, 2000; Soh, 2003; Stam and Elfring, 2008). Further, the feedback of these interactions and the anticipation of future interactions also enable both parties to develop social relations. Interpersonal trust in the competence, kindness, and integrity of others increases the desire to give and receive information, thus improving the performance of innovative collaborations (Chen and Hung, 2010; Duan et al., 2022).

### Cognitive Proximity and Innovation

Cognitive proximity refers to the similarities of the knowledge bases of actors (Nooteboom, 2000), which decide the extent of shared ability to perceive, comprehend, and evaluate the world among different actors (Knoben and Oerlemans, 2006). International business research has shown that cognitive distance often produces positive and negative effects simultaneously in knowledge diffusion and innovation (Reus and Lamont, 2009).

When there is a significantly similar knowledge base and know-how shared with actors, they will be more likely to cooperate as it makes their communication, learning, and sharing of knowledge more accessible (Marrocu et al., 2013; Pisoni, 2016). In this regard, cognitive proximity is essential for successful communication and knowledge spillover, which is considered as a primary condition for knowledge transfer and interactive learning (Mattes, 2012), promoting the formation and maintenance of cooperative relations, and improving collaborative innovation performance (Mancusi, 2008). Whereas continuous knowledge spillover is highly associated with absorptive capacity, since absorptive capacity enhances the impact of knowledge spillovers on knowledge creation (Camison and Fores, 2011), a firm’s innovation ability depends on the absorption capacity, especially for tacit knowledge, which is an essential factor for successful innovation and service performance. In this vein, significant cognitive proximity is also required in order to guarantee sufficient absorption of the capacity to identify, interpret and utilize new knowledge (Levinthal, 1990; Gilsing et al., 2008).

Contrary to the above-mentioned arguments, some literature addressed cognitive distance has more benefits for value creation purposes, as it allows for the recombination of heterogeneous knowledge inputs (Liu and Ma, 2019). Further, cognitive proximity can be detrimental to innovation because knowledge-creating also requires a degree of diversity and complementary knowledge base (Broekel et al., 2015). However, the marginal returns of adding further heterogeneity will decrease, simply because as the stock of heterogeneity of knowledge in an activity goes up, any knowledge encountered from further sources is less likely to be novel (Mol and Brandl, 2018).

### The Interactive Effects of Proximities on Knowledge-Intensive Business Service’ Innovation

There is evidence in the above systematic literature review that each dimension of proximity acts as a distinct enabler for learning and innovation. However, single proximity *per se* only explains part of the story. In fact, various forms of proximity strongly interact with one another within an innovative network, which is acknowledged as a comprehensive aspect to understand the knowledge flow underlying interactive learning and innovation (Mattes, 2012), especially when the knowledge is characterized by sticky, complex, or tacit. It is evident that single proximity is not enough to carry out all knowledge exchange among various actors in the process of interactive learning (Torre, 2008). In this vein, Boschma (2005) proposed that different dimensions of proximity inevitably interact with each other. For example, cognitive proximity may be associated with geographical proximity to affect the innovation performance of collaborations jointly. Therefore, the interaction effects of different proximity dimensions are significant for research in the fields of proximity, knowledge spillover, and innovation. Despite the attention received from researchers, extant studies generally mixed-up other kinds of proximities with geographical proximity to investigating their effects on service innovation. In this sense, we intend to propose how the interactions of the above-mentioned three dimensions of proximity influence the innovation performance of KIBS-client collaborations in the following part of this section to construct the main hypotheses.

### The Intersection Effect of Geographical Proximity and Social Proximity on Knowledge-Intensive Business Service’ Innovation

Previous literature claimed that geographical proximity and SP endorse similar roles, as they act as coordination and collaboration facilitators which all favor trust, commitment, and finally eases interactive learning (Cassi and Plunket, 2014). Many kinds of literature have proved that SP (but also organizational proximity and institutional proximity) most often happens in close geographical proximity because the latter contributes to facilitate and maintain the trust-based social networks, which further creates an environment that encourages knowledge sharing and effective collaboration (Koulikov, 2011). Geographical proximity thus plays a crucial role in improving innovation capacity through synergy and collective learning process in local (Balland, 2012).

Research on KIBS and innovation has highlighted the significant role of geographical proximity, whereas KIBS takes advantage of geographical proximity to establish social connections with their clients. Firstly, geographical proximity is crucial for KIBS organizations to develop continuous social contact with their clients, which helps them to understand the knowledge and capture the core requirements from their needs. It is common sense that a shared localized social network helps to enhance mutual trust, establish unified standards and attitudes regarding practical solutions, which is more conducive to interactive learning between customers and KIBS. Spatial proximity is also crucial to establish a broader learning process and further improve one’s status in the local social network (Menzel, 2015), hence, it is indispensable for KIBS to develop stable business relations for the tacit knowledge transfer between KIBS and clients requires frequent face-to-face communication with highly mutual trust.

Second, KIBS highly depends on the local innovation networks to reap desired benefits from proximity and accessibility. KIBS is largely contingent upon local innovation networks because localized collective learning has positive effects on KIBS firms (Nachum and Keeble, 2002). Nevertheless, new ideas derived from local areas are critical to stimulating KIBS’ innovative activities (Doloreux et al., 2010) since the unique competitive advantages of specific areas, regions, or cities contribute to the geographical agglomeration of firms, suppliers, service providers, and relevant institutions in particular industries (Porter, 1998). In this regard, actors interact with each other because of the externality and complementarity, which enables a larger scope of production coordination system within a various set of firms from different industries that build up production links of products and services. This, in turn, shapes the unique business climate in a specific region, which enables actors that have general localized knowledge and need to acquire new knowledge from the local environment, (Papanastassiou and Pearce, 1994; Zanfei, 2000). Nevertheless, the agglomerated environment promotes the innovation, learning, and sharing abilities of KIBS, which facilitates the dynamic process of boosting new innovative knowledge (Wood, 2002), which further makes it is easier for the service supplier and demander to match effectively.

Third, geographical proximity provides potential advantages in predicting the needs and opportunities for innovation. For example, it provides firms to monitor their competitors’ innovation actions, master the direction of market innovation in time, thereby taking the most proper innovative strategies (Porter and Stern, 2001). Hence, KIBS firms can master the changes of their clients’ needs instantly, consult the market trend, thereby cope with adjustments and improvements. To conclude, the interaction between geographical proximity and SP plays the role of collaborative facilitator, which is of great significance to the cooperation between KIBS and their clients, thereby promoting interactive learning, improving the collaborative innovation performance. Therefore, we put forward the following hypothesis:

*Hypothesis 1.* The intersection of geographical proximity and SP contributes to the knowledge exchange and absorption between KIBS and their manufacturing clients, thus improving the latter’s innovation performance.

### The Interaction Effect of Geographical Proximity and Cognitive Proximity on Knowledge-Intensive Business Service’ Innovation

When there is closer geographical proximity between the actors, face-to-face exchanges, and cooperation become more convenient, thereby promoting tacit knowledge transfer. Adversely, effective absorption of tacit knowledge requires a certain degree of cognition to facilitate effective communication and mutual trust, therefore promoting effective knowledge transfer and absorption. It has been well documented that knowledge flows generally happen between actors which are close in the cognitive proximity (cognitive proximity), while current cognitive proximity research has also emphasized its potential implications. Some scholars even claim cognitive proximity is one dimension of proximity, along with geographical proximity, which facilitates learning and knowledge creation (Hautala, 2011). It is relatively more difficult for actors which have similar technological bases in distant locations to understand and integrate knowledge because of the exorbitant cost of communication. On the contrary, firms in the same cluster may have more intensive shared specific technology-related knowledge, which further increases their knowledge with high over-lapping (Tanner, 2016). So, there is also a specific technological trajectory for knowledge to flow in geographically adjacent regions. Even in the case of radical technological development, the local environment is still important (Tanner, 2016).

In this sense, we believe that geographical proximity amplifies the effect of cognitive proximity on service innovation. The collaborative innovation based on a shorter geographic distance makes face-to-face communication more convenient, which therefore promotes tacit knowledge transfer and absorption (Broekel and Binder, 2007). Likewise, since KIBS firms can communicate with clients and receive feedback timely, the cognitive proximity will be more pronounced in an effective communication and coordination context. Similar technological backgrounds between KIBS and their clients are conducive to promoting the acquisition of heterogeneous resources, technology learning, and development, which contributes to integrating the complementary technological advantages between the two parties (Nesta and Saviotti, 2005). Moreover, geographical proximity helps find opportunities to collaborate with other actors that share a similar cognitive base before the technologies they mastered are outdated. Hence, KIBS can thus apply knowledge to clients more effectively.

Given that the interaction between cognitive proximity and geographical proximity can achieve better innovation performance, it is speculated that geographical proximity is necessary to capture the externalities that are derived from technical proximity. Hence, cognitive proximity that is often geographically close may result from the assimilation and interaction among firms. Therefore, we proposed the following hypothesis:

*Hypothesis 2.* The interaction of geographical proximity and cognitive proximity facilitates the knowledge exchange and absorption between KIBS and their manufacturing clients, thus improving the latter’s innovation performance.

### The Interaction Effect of Social Proximity and Cognitive Proximity on Knowledge-Intensive Business Service’ Innovation

There is a common belief that social and cognitive proximity intersections help actors to maintain the business relationships facilitating knowledge exchange (Frenkel et al., 2015). Actors with SP usually have better exchange for ideas and technological information, closer communication, and enhanced trust in social relations facilitate the identification and transfer of high-quality information which further increased their cognitive proximity while providing the common knowledge base that is necessary for cooperation (Breschi and Lissoni, 2005). Moreover, SP stimulates actors in collaboration to communicate on a mutual trust base, which will further improve the efficiency of collective learning, and finally enhance the impact of cognitive proximity on collaborative innovation performance. Cognitive proximity also reduces the barriers to collaboration while increasing the SP in the cooperation process (Balland, 2012). That is, when actors have a common history, interpersonal ties, and a particular technological background, they will have a greater propensity to establish collaboration and a higher possibility to develop successful innovation in collaboration.

It is noteworthy that service innovation requires a various set of knowledge, such as the customer preference, market demand, service process, business model, as well as new technology, etc. On the one hand, the core knowledge of KIBS is developed in specific service backgrounds according to their client’s needs and tacit in nature. In such circumstances, SP provides professional channels of knowledge dissemination, which means when there is frequent contact with a higher level of social trust- relationship, KIBS will be more willing to share knowledge with their clients (Howells, 2006), while cognitive proximity further increases the absorption efficiency of tacit knowledge. On the other hand, KIBS gets heterogeneous and complementary knowledge from social networks which help to establish an effective channel that provides for their clients to connect to useful external knowledge access across organizational boundaries (Muller and Zenker, 2001). Furthermore, when actors are close in the social networks, they can integrate a higher level of cognitive proximity to achieve positive effects. Hence, we developed the following hypothesis:

*Hypothesis 3.* The interaction of SP and cognitive proximity promotes the knowledge exchange and absorption between KIBS knowledge and their manufacturing clients, thus improving the latter’s innovation performance.

## Data Analysis

### Research Model

In this section, we will test the hypothesis proposed before. According to the empirical hypothesis mentioned above, we first constructed a structural equation as follows. Considering the essence of knowledge innovation (i.e., scientific discovery and technological invention) is the process of knowledge production, which could be embodied by constructing a KPF considering the input–output relationship of knowledge. In this sense, we take the knowledge flow from KIBS to the urban manufacturing industry in different urban areas as input factors that manufacturing firms conducting innovation activities. Thereafter, an input–output model based on the C-D function paradigm is constructed in Equation (1):


(1) $$l\,n\,P\,a\,t_{i\,t}=\alpha +\beta_{1}\,l\,n\,k\,i\,b\,s_{i\,t}+\beta_{2}\,C\,o\,n\,t\,r\,o\,l\,s+\varepsilon_{i\,t}$$

Where the dependent variable Pat_it_ proxies the level of INN in year t of the manufacturing industry in city *i*, which is represented by invention patent applications. The key independent variable lnINKIBS_it_ represents the knowledge that can be seen as a vital input for INN that KIBS has embedded into the manufacturing industry in city i in year t .*Controls* are the related control variables. As the usual practice, logarithmic processing is carried out for all independent variables, and ln(1 + *Pat*_*it*_) because there is some 0 record for dependent variables *Pat*.

According to the hypothesis proposed in section two and the SDM setting, we added two spatial spillover effects to the KPF model to depict the corresponding knowledge spillovers and therefore influence the latter’s innovation performance when considering different dimensions of proximities and their interaction effects. In this regard, it is of great significance to introduce the spatial weight factor, so the model is advanced as the following equation (2):

$$l\,n\,P\,a\,t_{i\,t}=\rho \cdot W\,\text{lnPat}+\beta_{1}\,l\,n\,I\,N\,K\,I\,B\,S_{i\,t}+\theta_{1}\cdot W\,l\,n\,I\,N\,K\,I\,B\,S_{i\,t}$$

$$+\beta_{2}\,C\,o\,n\,t\,r\,o\,l\,s+\theta_{2}\cdot W\,C\,o\,n\,t\,r\,o\,l\,s+u$$

(2) u=γ⋅W⁢u+ε

Where the matrix W is the preset spatial weight matrix, *WlnPat*presents the endogenous interaction between the dependent variables, which means that there is a spatial correlation between the INN of a specific region and that of the other neighboring regions; _WlnINKIBS*it*_indicates the knowledge flow absorbed by the manufacturing industry in city i from KIBS in other cities to increase the exogenous effect of IN. Wu is the interaction effect between the interference terms of different space observation units, that is, the interaction effect between the error terms.

### Data and Variables

The panel data in this study are derived from the China Industry Business Performance Database, the China Patent Database, and the China City Statistics Database, which covered 260 cities at the prefectural level in China from 2003 to 2014. It should be noted that because of the missed data, the research sample does not include Hong Kong Macao, Taiwan, as well as some regions of Xinjiang, Tibet, Hainan, Inner Mongolia, Gansu, Yunnan, etc.

The dependent variable is the innovation performance of the manufacturing industry at the city level. We measure it by the number of invention patent applications of a city’s manufacturing industry. It should be noted that data regarding patents usually refers to the number of patent applications and patent grants. Furthermore, patents are divided into three types, which are invention patent, utility model patent, and design patent, respectively. In particular, the invention patent has relatively high technical content and low application volume, which therefore will be less constrained by the examination ability of patent authorizing agency. In this vein, it is clear that invention patents could be more objective to reflect the originality and quality regarding the innovation capability of manufacturing industries in a specific region.

The key independent variable reflects expected knowledge transferring from KIBS to manufacturing firms in a specific sector located in a specific city. Specifically, we calculate the KIBS input of manufacturing industries at the industry level, then measure the KIBS input of manufacturing output at the firm level from the perspective of output. The calculation can be divided into two steps, first, we outline the equation as (3) shows:


(3) $${\text{kibs}}_{\text{jt}}=\sum_{\text{m}=1}^{4}\frac{{\text{kibs}}_{\text{mjt}}}{{\text{y}}_{\text{jt}}}$$

Where m indexes the certain industries of KIBS, whereas *m* = 4, in this study we include 4 representative KIBS industries in our study (information transmission, software and information technology services; the financial sector; scientific research and technology services; leasing and business services), j represents the specific manufacturing industry; So kibs_mjt_ indicates the knowledge transferred from KIBS m to manufacturing industry j in year t. y_jt_ Indicates the total input of manufacturing j in year t, which can be measured by multiplying the annual output of j by the proportion of intermediate input to the total output of itself.

Second, we further calculate the


(4) $${\text{kibs}}_{\text{it}}=\frac{{\text{output}}_{\text{ijt}}}{\sum_{\text{i}}{\text{output}}_{\text{ijt}}}\times {\text{kibs}}_{\text{jt}}$$

Where output_ijt_ is the industrial output of firm i in the manufacturing industry, j in year t ; _∑i_output_ijt_ is the total industrial output of the manufacturing industry j over the years;_INKIBSjt_ depicts the degree that the intermediate input of KIBS in the manufacturing industry aggregation, which is measured by the proportion of the KIBS input in the total input of manufactory sectors. Finally, we summed up the relevant data at the regional level in the China Industry Business Performance Data, the manufacturing industry output in a region containing KIBS input is obtained.

In addition to the key independent variable, other control variables are as follows:

(1) R&D expenditure (*lnrd*): we measure R&D expenditure as a city’s R&D expenditure where the manufacturing firm is located. To be more specific, we used the price indices of investment in 2003 to subtract the R&D expenditure and then achieved the modified R&D expenditure at the city level in 2003.
(2) Human capital input (*lnhum*): we measure human capital input in this study by the number of college students per 10,000 people in a city.
(3) Physical capital input (*lnperk*): It is depicted by the physical capital per capita, which is calculated by the ratio of fixed capital stock to the population of a city.
(4) Foreign direct investment (*lnfdi*). It measured by the ratio of the actual amount of foreign direct investment to a city’s GDP.
(5) Manufacturing employees (*maf*): It is measured by the proportion of manufacturing employment in total employment in the city.
(6) Environmental pollution (*Inpoll*): It is expressed by the amount of investment in environmental pollution control.
(7) Population density (*Inpop*): It is calculated by the population divided by urban area (square kilometers).
(8) Transportation infrastructure (*Inway*): It is expressed by highway mileage.

The Descriptive statistics of each variable are shown in the Table 1.

**TABLE 1:** Descriptive statistics of variable.

Variable	*N*	Mean	*SD*	Min	Max
*Inpat*	3120	1.18	0.872	0	4.174
*lnkibs*	3120	0.386	0.142	–1.31	0.679
*ln*rd	3120	–1.27	0.257	–2.351	–0.544
*Inhum*	3120	1.804	0.559	–.905	3.104
*Inmaf*	3120	–0.655	0.248	–2.034	–0.09
*Inpoll*	3120	4.44	0.76	0	8.544
*Inpop*	3120	2.522	0.367	1.001	3.425
*Inway*	3120	3.659	0.512	0	5.291
*lnfdi*	3120	–0.295	0.374	–1.303	0.365
*lnper*k	3120	–0.013	0.528	–1.43	1.365

### The Measurements of Different Spatial Weight Matrixes

According to the hypotheses proposed in Section “Theoretical Basis and Research Hypothese,” we select three spatial weight matrixes which indicate geographical proximity, cognitive proximity, and SP, respectively, and the measurement of each matrix is explained as follows:

(1) The weight matrix for geographical proximity

We used Matlab to calculate the spherical distance (*d*_*ij*_) between any two cities and the longitude and latitude coordinates of 260 prefecture-level cities were obtained from the website of the national Geographic Information System. The specific geographical proximity index is expressed as $\frac{1}{d_{i\,j}^{2}}$. The larger the distance between two districts, the smaller the index value, indicating that there is lower geographical proximity between two districts; conversely, the smaller the distance between two districts, the larger the index value, indicating that there is higher geographical proximity between two districts. The geographical proximity weight matrix is set as equation (5):


(5) $$W_{i\,j}^{d}=\left\{ \begin{array}{cc} \frac{1}{d_{i\,j}^{2}}, & i\neq j\\ 0, & i=j \end{array}\right.$$

(2) The weight matrix for cognitive proximity

We use the Jaffe (1989) index to measure the cognitive proximity between different cities. The calculation formula is as follows:


(6) $$W_{i}^{c}=\frac{\sum_{k=1}^{8}F_{k\,i}\,F_{k\,j}}{\sqrt{\sum_{k=1}^{8}F_{k\,i}^{2}\,\sum_{k=1}^{8}F_{k\,j}^{2}}}$$

Where *k*represents different technical sectors of invention patent applications, *k* 1……8 (A-Necessities of human life; B-homework; Transport; C - chemistry; Metallurgy D-textile; papermaking; E-Fixed buildings; F-Mechanical Engineering; Lighting; Heating; Weapons; Blasting; G - Physical; H- Electrical). *i* and *j* represent different cities, whereas *i*≠j.*F*_*ki*_represents the number of invention patents generated by city*i*in the technology field*k*. The value of this formula is between 0 and 1, which means, the larger the value is, the higher the cognitive proximity between the two cities; the closer it is to 0, the lower the cognitive proximity between the two cities. We use the index to construct the adjacency matrix directly at the corresponding position of the matrix.

(3) The weight matrix for social proximity

We adopted the Jaccard index to measure the intensity of cooperative scientific research publications, which indicates social proximity in innovation between different cities. The calculation is as follows:


(7) $$W_{i\,j}^{s}=\frac{\gamma_{i\,j}}{\gamma_{i,\cdot }+\gamma_{\cdot j}-\gamma_{i\,j}},\left(i,j=1,2,\ldots ,n\right)$$

$$\gamma_{i,\cdot }=\sum_{j=1,i\neq j}^{n}\gamma_{i\,j},\gamma_{\cdot j}=\sum_{i=1,i\neq j}^{n}\gamma_{i\,j}$$

γ_*ij*_ is the number of cooperative papers between city *i* and city *j*; γ_*i*⁣⋅_ and _γ⋅*j*_ are the sum of the papers co-authored in city *i* and city *j*, respectively.

(4) Nested matrixes for interactions of different dimensions of proximity

In order to test the intersection effects of different dimensions of proximity as proposed by the hypotheses in Section “Theoretical Basis and Research Hypothese,” we compute three nested matrices as follows:


(8) Wi⁢jx⁢x=′ϕWi⁢jx+(1-ϕ)Wi⁢jx′

Where *W^d*_*ij*_ indicate a weight matrix of geographical proximity. $W_{i\,j}^{x}$ represents a nested weight matrix for non-geographical proximity. Where *x*≠*x*′ can indicate social proximity, cognitive proximity, and geographical proximity, respectively. In accordance with practice, we set the value of ϕ = 0.5. The nested matrix of geography considers the intersections of geographical proximity and non-geographical proximity. In this study, the geographical and social nested matrices can be expressed by $W_{i\,j}^{d\,s}$, the cognitive and social nesting matrix is $W_{i\,j}^{c\,s}$, and the cognitive and geographical nesting matrix is $W_{i\,j}^{d\,c}$.

## Empirical Test

### Moran’s Index for Dependent Variable

Before the regression analysis, we first obtained the Moran’s I for the dependent variable to check whether the invention applications of the manufacturing industry in 260 Cities in China from 2003 to 2014 with different three proximity weight matrices, respectively. As shown in Table 2, we can see that the dependent variables show significant positive spatial autocorrelation. Moreover, the coefficients of autocorrelation of *lnpat*under geographical proximity are ever-increasing from 2003 to 2014, and slightly decreases under cognitive proximity and SP from 2012 to 2014, which shows the urban manufacturing INN is not random in spatial distribution, the city with higher INN tends to be adjacent to other cities with higher INN, that is, high-high agglomeration.

**TABLE 2:** Moran’s *I*-value for lnpat under different weight matrixes.

Year	Geographical proximity	Cognitive proximity	Social proximity
2003	0.127***	0.025***	0.332***
2004	0.170***	0.026***	0.350***
2005	0.220***	0.025***	0.373***
2006	0.219***	0.025***	0.343***
2007	0.232***	0.028***	0.354***
2008	0.265***	0.031***	0.369***
2009	0.284***	0.031***	0.395***
2010	0.304***	0.032***	0.407***
2011	0.309***	0.032***	0.432***
2012	0.289***	0.029***	0.380***
2013	0.283***	0.027***	0.381***
2014	0.310***	0.026***	0.390***

*** indicates statistical significance at the 1% level.

### The Regression Results

**FIGURE 1:**
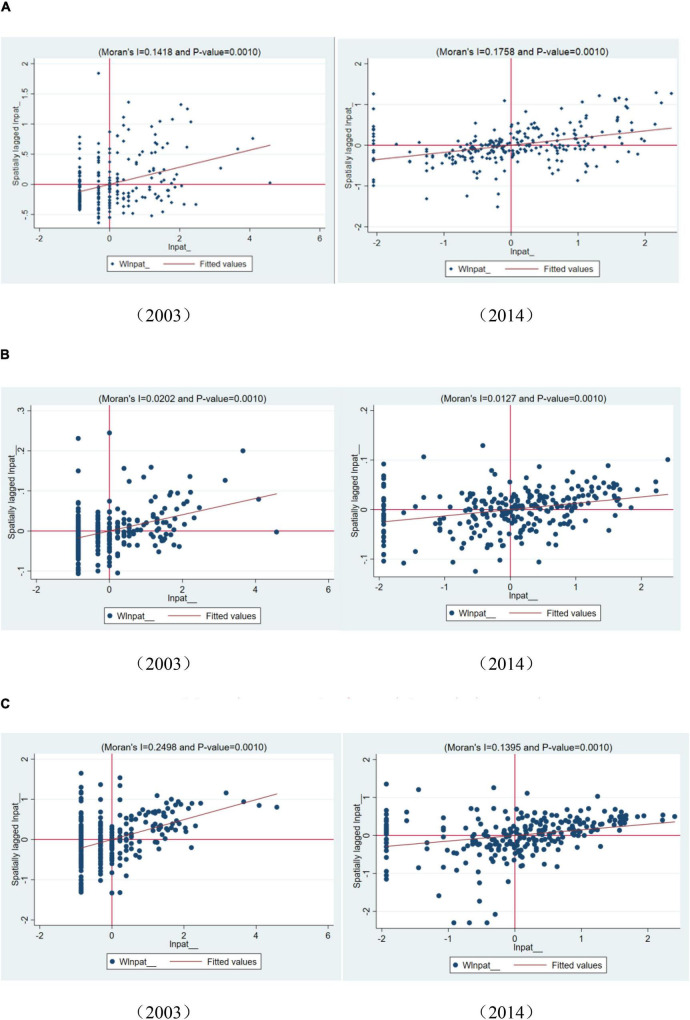
Moran’s scatter plot for geographic, social, and cognitive matrixes. **(A)** Moran’s scatter plot for geographical proximity matrix. **(B)** Moran’s scatter plot for cognitive proximity matrix. **(C)** Moran’s scatter plot for social proximity matrix.

Considering there may exist the endogenous problem of the dependent variable and key independent variables, we adopted a fixed-effect space regression model with panel data to conduct empirical analysis. In addition, we selected the best spatial model fit for our sample data. It is shown that the LR tests for SDM and SEM are 147.61, the LR test for SDM and SAR are 131.77, and the coefficients of which all passed the significance test at the 1% level, rejecting the null hypothesis. Hence, it is concluded that Spatial Durbin Model (SDM) is optimal when compared with the spatial lag model fitting effect (SAR) and the spatial error model (SEM). In addition, the coefficient of Wald tests is 110.35 and significant at the 1% level, which further indicates that the spatial-temporal double-fixed Durbin model failed the Wald and LR tests in terms of the geographical, social, and technological weight matrix. They can neither be converted into the SAR model nor the SEM model. We supplemented the Moran Scatter plot for the geographical, social, and technological weight matrixes in Figure 1. Then, combined with the maximum likelihood value (log-L) and the adjusted determination coefficient (*R*^2^), we adopt the spatial Durbin model (SDM) with fixed effects. To better interpret the influence effect of coefficients, we further decomposed the coefficients into the direct effect, the indirect effect and the total effect by partial differential effect calculations. Whereas the direct effect indicates the direct influence of independent variable on its own dependent variable, while the indirect effect indicates the spatial spillover effect of independent variable on the dependent variable from neighboring cities, and the total effect is the integrate effect of direct effect and indirect effect.

### The Impacts of Single Dimension of Proximity on the Innovation Performance of Knowledge-Intensive Business Service – Manufacturing Collaboration

Table 3 shows how different dimensions of proximity influence the innovative performance of KIBS-manufacturing collaboration individually. The regression results have supported what we have discussed in the literature review part for how different dimensions of proximities act on the collaboration between different organizations.

**TABLE 3:** The innovation performance of knowledge-intensive services (KIBS)-manufacturing collaboration under a single dimension of proximity.

	Geographical proximity			Social proximity			Cognitive proximity		
Variable	Direct	Indirect	Total	Direct	Indirect	Total	Direct	Indirect	Total
lnkibs	0.0705	0.5841**	0.6546***	0.1496	0.6827**	0.8323***	0.1526***	0.1223	0.2748
	(0.481)	(0.028)	(0.009)	(0.129)	(0.010)	(0.001)	(0.000)	(0.789)	(0.553)
lnrd	0.1848***	1.9729***	2.1577***	0.2951***	0.9301***	1.2252***	0.1583***	0.1305	0.2888
	(0.000)	(0.000)	(0.000)	(0.000)	(0.000)	(0.000)	(0.000)	(0.692)	(0.384)
lnhum	0.0177	−0.1668*	−0.1492*	−0.0003	0.0080	0.0077	0.0123	0.0087	0.0210
	(0.579)	(0.061)	(0.079)	(0.992)	(0.915)	(0.919)	(0.315)	(0.870)	(0.711)
lnmaf	0.1460**	0.4747*	0.6207**	0.2933***	0.4540**	0.7473***	0.1251***	0.1037	0.2288
	(0.028)	(0.056)	(0.014)	(0.000)	(0.032)	(0.001)	(0.000)	(0.711)	(0.424)
lnpoll	−0.0071	0.0164	0.0092	0.0100	0.0712***	0.0812***	0.0040	0.0034	0.0075
	(0.481)	(0.690)	(0.827)	(0.318)	(0.009)	(0.006)	(0.372)	(0.866)	(0.731)
lnpop	−0.0924	−0.2359	−0.3283	−0.0834	−0.4384	−0.5218*	−0.0650*	−0.0532	−0.1182
	(0.334)	(0.506)	(0.332)	(0.394)	(0.160)	(0.075)	(0.095)	(0.761)	(0.526)
lnhway	0.0014	0.4369***	0.4382***	0.0158	0.0150	0.0308	0.0126	0.0122	0.0248
	(0.952)	(0.000)	(0.000)	(0.485)	(0.802)	(0.620)	(0.372)	(0.779)	(0.620)
lnfdi	−0.0488	0.3035	0.2547	−0.0604*	0.1325	0.0722	−0.0351***	−0.0292	−0.0643
	(0.124)	(0.147)	(0.243)	(0.059)	(0.164)	(0.480)	(0.001)	(0.754)	(0.498)
lnperk	0.1520**	−0.2053	−0.0533	0.1214**	−0.4372***	−0.3157*	0.0189	0.0187	0.0376
	(0.014)	(0.362)	(0.812)	(0.044)	(0.004)	(0.052)	(0.121)	(0.524)	(0.504)
Year	Control	Control	Control	Control	Control	Control	Control	Control	Control
City	Control	Control	Control	Control	Control	Control	Control	Control	Control
ρ	0.3076***	0.3076***	0.3076***	0.2192***	0.2192***	0.2192***	0.6013***	0.6013***	0.6013***
Obs	3,120	3,120	3,120	3,120	3,120	3,120	3,120	3,120	3,120
*R* ^2^	0.434	0.434	0.434	0.260	0.260	0.260	0.327	0.327	0.327

P-value in parentheses, “***”, “**”, “*” indicate statistical significance at the 1, 5, and 10% level.

First, in the case of geographical proximity, the indirect effect of KIBS’ knowledge flow to the manufacturing industry on the latter’s innovation performance is significantly positive, which indicates that the spatial collaboration between KIBS and the manufacturing industry is beneficial to increase complementary knowledge transfer and stimulate the exchange of innovative ideas. As can be seen from the indirect effect, geographical proximity also contributes to the transfer and communication of innovative ideas and knowledge such as information and technology from KIBS to manufacturing industries located in different cities, and further facilitates the manufacturing industry to acquire knowledge and innovative ideas from KIBS in different cities, and thus producing a positive promotion to its INN.

Second, in the case of social proximity, the indirect effect of the degree of interaction between KIBS and manufacturing passed the significance test of 5% and the sign was positive. This regression also shows that social proximity intensifies the interaction between KIBS and the manufacturing industry positively, and thus promotes the improvement of the innovative output of the latter. This regression also indicates that social proximity is at least as important as geographical proximity for knowledge transferring between actors. In the case of social proximity, mutual trust in the social network provides channels for tacit knowledge dissemination, which is strengthened in the interactive learning between KIBS and the manufacturing industry. Thus, positive knowledge spillover effect is generated in cities with close social connections.

Third, in the case of cognitive proximity, KIBS has a positive and significant direct effect on local manufacturing innovation performance, but the indirect effect is not significant. The results indicate that the knowledge spillover between KIBS and the manufacturing industry is difficult to produce significant innovation promotion effects based on cognitive proximity without coordination mechanisms such as geographical proximity or social proximity.

### The Impacts of Proximity Interactions on the Innovative Performance of Knowledge-Intensive Business Service – Manufacturing Collaboration

#### The Regression Results of Core Independent Variables

According to the three hypotheses which have been proposed in Section “Theoretical Basis and Research Hypothese,” we conducted regression analysis on equation (2), which test the effects of geographical proximity, cognitive proximity, and SP interact in pairs on the innovation performance of KIBS-manufacturing collaboration, and the regression results are shown in Table 4.

**TABLE 4:** The regression tests under different proximity’s intersection weight matrix.

	Geographical and social proximity			Geographical and cognitive proximity			social and cognitive proximity		
Variables	Direct	Indirect	LR_Total	LR_Direct	LR_Indirect	LR_Total	LR_Direct	LR_Indirect	LR_Total
lnkibs	0.0725	0.6197*	0.6922**	0.0713	1.9029**	1.9743**	0.1440	1.9497**	2.0936***
	(0.523)	(0.071)	(0.026)	(0.473)	(0.031)	(0.021)	(0.147)	(0.011)	(0.005)
lnrd	0.2603***	1.7904***	2.0508***	0.1979***	6.4402***	6.6382***	0.3070***	2.2291***	2.5362***
	(0.000)	(0.000)	(0.000)	(0.000)	(0.000)	(0.000)	(0.000)	(0.000)	(0.000)
lnhum	0.0316	−0.1702	−0.1386	0.0191	−0.5609**	−0.5419*	0.0094	−0.0950	−0.0856
	(0.402)	(0.115)	(0.168)	(0.547)	(0.049)	(0.051)	(0.750)	(0.641)	(0.672)
lnmaf	0.0625	1.0107***	1.0732***	0.1481**	1.4418*	1.5899**	0.2792***	1.4533**	1.7325***
	(0.428)	(0.001)	(0.001)	(0.026)	(0.067)	(0.043)	(0.000)	(0.015)	(0.004)
lnpoll	−0.0064	0.0379	0.0315	−0.0067	0.0394	0.0327	0.0142	0.2045***	0.2188***
	(0.591)	(0.393)	(0.486)	(0.508)	(0.756)	(0.797)	(0.160)	(0.009)	(0.006)
lnpop	0.0008	−0.7031	−0.7023*	−0.0914	−0.7780	−0.8695	−0.0621	−1.6090*	−1.6711**
	(0.994)	(0.124)	(0.092)	(0.335)	(0.486)	(0.426)	(0.528)	(0.067)	(0.050)
lnhway	−0.0214	0.1739*	0.1525	0.0050	1.3386***	1.3435***	0.0102	0.0210	0.0311
	(0.430)	(0.087)	(0.125)	(0.827)	(0.000)	(0.000)	(0.658)	(0.900)	(0.852)
lnfdi	−0.0572	0.0501	−0.0071	−0.0466	0.8883	0.8417	−0.0584*	0.2938	0.2354
	(0.126)	(0.786)	(0.970)	(0.143)	(0.177)	(0.206)	(0.070)	(0.263)	(0.377)
lnperk	0.0939	−0.2556	−0.1617	0.1478**	−0.6327	−0.4850	0.1580***	−2.3409***	−2.1829***
	(0.205)	(0.335)	(0.529)	(0.016)	(0.367)	(0.485)	(0.010)	(0.000)	(0.000)
Year	Control	Control	Control	Control	Control	Control	Control	Control	Control
City	Control	Control	Control	Control	Control	Control	Control	Control	Control
ρ	0.2070***			0.5364***			0.4076**		
Observations	3,120			3,120			3,120		
*R*−squared	0.414			0.320			0.211		

P-value in parentheses, “***”, “**”, “*” indicate statistical significance at the 1, 5, and 10% level.

(1) The interaction of geographical proximity and social proximity weight matrixes has a positive effect on the innovation performance of KIBS-manufacturing collaboration, which means geographical proximity and SP have similar coordination mechanisms. Knowledge transfer will not only decrease efficiency due to the increase of geographical distance but also incur higher communication costs due to the alienation of social relations. Therefore, the knowledge spillover caused by the interaction between KIBS and the manufacturing industry needs both geographical proximities to maintain frequent and low-cost interactions and SP to establish close social connections. There is a complementary relationship between geographical proximity and SP. Geographical proximity facilitates social interaction and forms social networks, thus strengthening SP. Therefore, under the interaction of geographical proximity and SP, the interaction between KIBS and the manufacturing industry can produce spillover effects, supporting Hypothesis 1.
(2) The interaction of geographical proximity and cognitive proximity has a positive effect on the innovation performance of KIBS-manufacturing collaboration. Cognitive proximity, which was not significant originally, became significant after interaction with geography, indicating a positive sign. Specifically, although the interaction between KIBS and the manufacturing industry has no significant impact on the INN of adjacent regions under the single cognitive proximity, it will have a positive spillover effect under the interaction of geographical proximity and cognitive proximity. It can be considered that although enterprises with single cognitive proximity have a similar knowledge base, there is no spillover path due to the lack of technical knowledge of communication media, and the degree of technology spillover is indeed highly dependent on geographical distance. However, under the interaction of geographical proximity and cognitive proximity, geographical proximity can reduce communication costs and promote the transmission and absorption of tacit knowledge, and cognitive proximity can lay a knowledge foundation for effective communication between the two sides. Moreover, firms with similar technical backgrounds are more inclined to seek complementary technical resources within the geographical range of adjacent. Therefore, the combination of geographical proximity and cognitive proximity is more conducive to improving innovation performance, which supports Hypothesis 2.
(3) The interaction of social proximity and cognitive proximity has a positive effect on the innovation performance of KIBS-manufacturing collaboration, which is similar to the regression result of the intersection with geographical proximity. The coefficient for indirect effect is also significant at the 5% level. It can be speculated that although cognitive proximity cannot achieve knowledge spillover, social proximity can build a relationship network of mutual trust for both parties, which provides channels for the dissemination of professional knowledge, and improve organizational communication efficiency. While cognitive proximity increases the absorption efficiency of professional knowledge, the combination of the two promotes knowledge spillovers and increases INN, supporting Hypothesis 3.

#### The Regression Results of Other Control Variables

Among the control variables, the influence of R&D input on INN is significant in the three weight matrices, and the direct effect and indirect effect pass the significance test of 1%. This indicates that R&D investment not only promotes local innovation performance significantly but also has a positive spillover effect on surrounding areas. This is consistent with the finding of most studies that the more research and development is spent, the more innovation is likely to result. The only indirect effect of human capital investment passed the significance test of 10 and 5% in the geographic matrix and cognitive matrix, respectively, and the other effects were not significant. This may be because the number of university students is used as the measurement index of human capital investment. College students have high education levels but which is not accurately reflect the real skilled worker stock living in cities, and they prefer to choose cities near their universities or hometown. As a result, human capital investment has a significant indirect effect on geographical proximity and cognitive proximity level. The spillover effect of industrial structure on INN is significant in all three matrixes, which indicates that the size of the manufacturing industry will promote INN, especially the positive spillover effect between firms with similar technology. The flow of professional talents or skilled workers promotes the improvement of manufacturing INN in the same professional field. The degree of environmental pollution only has a significant positive effect on the INN of the adjacent regions, which may be related to the parent company retaining the core high-tech industry and transferring the high-pollution and low-skill industry to the subsidiary. Only in the social matrix, population density has a significant negative total effect, indicating that in the case of close social relations, high population density may be detrimental to INN. The indirect effects of transport infrastructure pass the significance test of 1% in both geographical and cognitive matrices. In the case of geographical proximity, the convenience of transportation infrastructure promotes the free flow of human capital between cities, which has a positive effect on the INN of surrounding regions. Because actors prefer to choose to cooperate with other actors with a large technological gap, transportation infrastructure has a restraining effect on the INN of neighboring regions. Foreign direct investment (FDI) has a significant negative direct effect on regional INN only in the social matrix, which indicates that competition effect and crowding effect caused by FDI inhibit local innovation. The direct effect of material capital input passes the significance test of 5% in all three matrices, while the indirect effect is significant and negative only in the social matrix. In this paper, the per capita fixed capital of a city is used to represent the material capital input. The development level of a city has a siphonic effect on surrounding cities and inhibits the INN of nearby cities.

### Further Test

**FIGURE 2:**
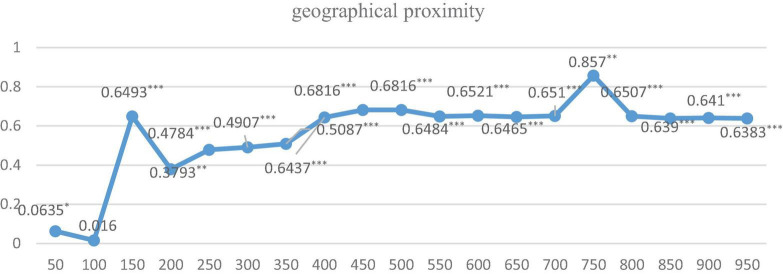
The regression results under geographical proximity with different kilometers. ***, **, and * indicate that the significance level is at the 1%, 5%, and 10%, respectively.

**FIGURE 3:**
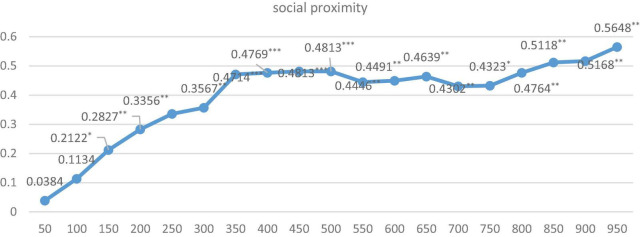
The regression results under social proximity with different kilometers. ***, **, and * indicate that the significance level is at the 1%, 5%, and 10%, respectively.

**FIGURE 4:**
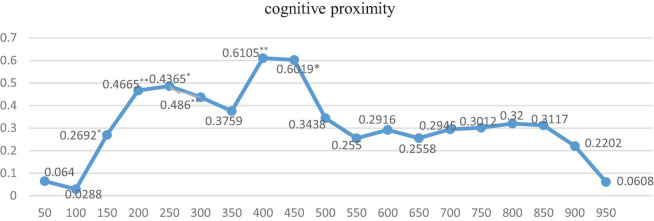
The regression results under cognitive proximity with different kilometers. ** and * indicate that the significance level is at the 5% and 10%, respectively.

Furthermore, in order to explore the optimal spatial range under the above-mentioned 3 spatial weight matrixes, respectively, and their two pair’s intersection settings, we further run the regression by dividing the distance of cities into 50 km units, ranging from 0 to 1,000 km. Just as Equation 2, we first give the indirect coefficients and their significance level under three dimensions of proximity is shown in Figures 2–4.

As it is shown in Figures 2, 3, the indirect effects under geographical matrix and social matrix alone are all positive and significant during 1,000 km, except for coefficients within 0–100 km, and coefficients for these two matrices are approximate around 0.6 for geographic matrix and above 0.4 for the social matrix on average if the spatial range is more than 350 km and relatively stable. Differently, the indirect effects under the cognitive matrix shown in Figure 3 are significant only in the range of 150–450 km, indicating that the diffusion of KIBS tacit knowledge is strongly restricted by geographical boundaries. Once beyond 450 km, single cognitive proximity makes it difficult for tacit knowledge to spread. The regression from Figures 2–4 also indicates that it is necessary to build effective communication channels by means of geographical proximity or society to realize knowledge spillover.

**FIGURE 5:**
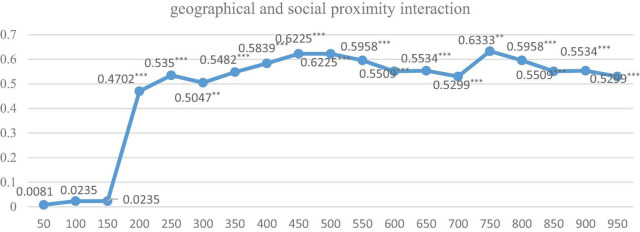
The regression results under geographic and social weight matrix with different kilometers. *** and ** indicate that the significance level is at the 1% and 5%, respectively.

**FIGURE 6:**
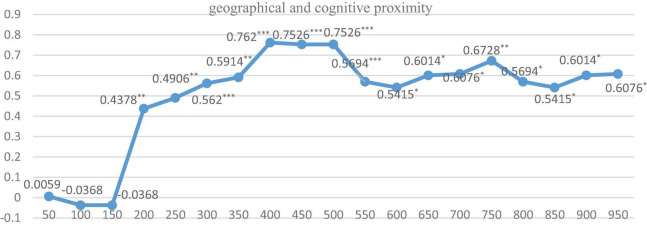
The regression results under geographic and cognitive weight matrix with different kilometers. ***, **, and * indicate that the significance level is at the 1%, 5%, and 10%, respectively.

**FIGURE 7:**
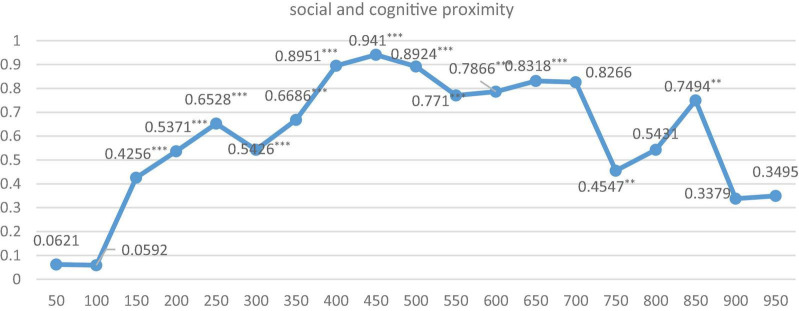
The regression results under cognitive and social weight matrix with different kilometers. *** and ** indicate that the significance level is at the 1% and 5%, respectively.

The regression results of nested matrixes for the three pairs of intersections of these proximity dimensions (i.e., geographic-social, geographic-cognitive, and social-cognitive) are shown in Figures 5–7. It is indicated that when geographical proximity intersects with social proximity and exceeds 200 km, all indirect effects are positive and significant. While when cognitive proximity intersects with geographical proximity or social proximity, the level of significance rises dramatically, significant from 200 to 600 km. It can be speculated that the scope of knowledge spillover is expanded under the interaction effect, and geographical proximity and social proximity build knowledge transmission channels between KIBS and the manufacturing industry with similar foundations.

The regression with different spatial scope intervals shows similar results and further proves our findings in Section “The Regression Results,” which can be seen as a robust test of our empirical study.

## Conclusion

There is a growing number of studies on how different dimensions of proximity and their interactions exert significant knowledge diffusion or spillover effects, which then could impact the innovation performance of inter-organizational collaborations. However, it is noted that this subject remains in the exploring stage that deserves further discussion. This study extends the frontier research of service innovation and organization-collaboration by providing deeper insight into the influencing mechanism of different proximity dimensions (i.e., geographic, cognitive, and social proximity) affecting the efficiency of knowledge flows from KIBS to their manufacturing clients, which incorporated the co-location or geographical proximity effects in the service-related study. Moreover, this study is conducive to unlocking the black-box of the interactive learning behaviors between knowledge-based services and the real economic sectors.

Specifically, three pairs of intersections of different proximity dimensions (i.e., geographic-social, geographic-cognitive, and social-cognitive) are analyzed, which may profoundly impact the knowledge transfer between KIBS and manufacturing firms, thereby influencing the latter’s innovation performance. We empirically quantified the corresponding effects by an SDM and applied them to a panel data set that covers 260 Chinese cities at the prefectural level and above from 2003 to 2014. The major findings of this study are as follows:

(1) In the innovation collaboration between KIBS and the manufacturing industry, geographical proximity, and SP have similar effects. Either geographic or social proximity contributes to reducing the cost to search for collaboration partners, building mutual trust within social networks in the collaboration process, and promoting interaction between KIBS and their manufacturing clients. In this sense, the intersection between geographical proximity and social proximity promotes interactive learning, which helps to build up a bridge for the tacit knowledge exchange between KIBS and its clients.
(2) Cognitive proximity provides collaborations for different organizations with a similar knowledge base, which enhances the communication efficiency in the processes regarding knowledge transfer and the exchange of innovative ideas, thus promoting optimal innovation outcomes. However, without the geographical proximity or SP, the implications of cognitive proximity in interactive learning will be significantly diminished, considering there will be a shortage of opportunities for effective interaction among collaboration partners.
(3) The spatial range of KIBS knowledge spillovers under different proximity dimensions are further investigated in this study, and the empirical results indicate that the effective spatial range of geographical proximity and social proximity is significant within 1,000 km, respectively. Adversely, the effective spatial range for cognitive proximity is significant only within 450 km. In addition, when interacting with geographical proximity and social proximity, the effective space range of cognitive proximity is expended to 1,000 and 600 km, respectively. It is concluded that the scope of cognitive proximity is greatly influenced by the coordination mechanism of geographical proximity and social proximity, suggesting cognitive proximity effectively breaks through the geographical boundaries to promote knowledge exchange.

## Limitations and Future Research Directions

Although in this study, we verify how different proximities and their interactions contribute to the diffusion of knowledge from KIBS to manufacturing and thus impact the innovation performance of the latter differently. It is evident that this study is of great significance in producing several contributions to KIB research and policy inspirations. Clearly, it is not without limitations. First, the interactive learning between KIBS and manufacturing is more complex and dynamic, as the effect of interactive connections between KIBS and their clients is not simply the sum of knowledge spillover of all KIBS providers from different locations. The optimal combination level of proximities that the whole knowledge network provides various intact knowledge to all partners with different proximity under the joint action of different actors. Second, it is clear that the geographic, cognitive, and social proximities may also be strengthened by the experience of cooperation between actors. Moreover, KIBS and their clients with high innovative capacity can be more initiative to expand the network of cooperation, which will enhance the proximity of all dimensions with KIBS, thus future studies can focus on the complexity of the knowledge exchange process between KIBS and clients, which can further employ the heterogeneity of clients and go deeper to study specific innovation activities sensitivity to different proximities.

## Data Availability Statement

The datasets presented in this study can be found in online repositories. The names of the repository/repositories and accession number(s) can be found in the article/supplementary material.

## Author Contributions

TZ was responsible for writing—original draft, formal analysis, methodology, and conceptualization of this study. MY contributed to writing—original draft, resources, conceptualization, and proofreading. ZC assisted in resources, writing—review and editing, and validation. XW made efforts in writing—review and editing. All authors contributed to the article and approved the submitted version.

## Conflict of Interest

The authors declare that the research was conducted in the absence of any commercial or financial relationships that could be construed as a potential conflict of interest.

## Publisher’s Note

All claims expressed in this article are solely those of the authors and do not necessarily represent those of their affiliated organizations, or those of the publisher, the editors and the reviewers. Any product that may be evaluated in this article, or claim that may be made by its manufacturer, is not guaranteed or endorsed by the publisher.
